# A novel duplication polymorphism in the *FANCA *promoter and its association with breast and ovarian cancer

**DOI:** 10.1186/1471-2407-5-43

**Published:** 2005-04-29

**Authors:** Ella Thompson, Rebecca L Dragovic, Sally-Anne Stephenson, Diana M Eccles, Ian G Campbell, Alexander Dobrovic

**Affiliations:** 1 Department of Pathology, Peter MacCallum Cancer Centre, Locked Bag 1 A'Beckett St, Melbourne, Victoria 8006, Australia; 2 University of Adelaide Department of Medicine and Department of Haematology/Oncology, The Queen Elizabeth Hospital, Adelaide, South Australia 5011, Australia; 3 Wessex Clinical Genetics Service, Princess Anne Hospital, Southampton, SO16 5YA, UK; 4 Centre for Genomics and Predictive Medicine, Peter MacCallum Cancer Centre, Locked Bag 1, A'Beckett St, Melbourne, Victoria, 8006, Australia; 5 Department of Pathology, University of Melbourne, Parkville Victoria, 3002, Australia

## Abstract

The *FANCA *gene is one of the genes in which mutations lead to Fanconi anaemia, a rare autosomal recessive disorder characterised by congenital abnormalities, bone marrow failure, and predisposition to malignancy. *FANCA *is also a potential breast and ovarian cancer susceptibility gene. A novel allele was identified which has a tandem duplication of a 13 base pair sequence in the promoter region.

**Methods:**

We screened germline DNA from 352 breast cancer patients, 390 ovarian cancer patients and 256 normal controls to determine if the presence of either of these two alleles was associated with an increased risk of breast or ovarian cancer.

**Results:**

The duplication allele had a frequency of 0.34 in the normal controls. There was a non-significant decrease in the frequency of the duplication allele in breast cancer patients. The frequency of the duplication allele was significantly decreased in ovarian cancer patients. However, when malignant and benign tumours were considered separately, the decrease was only significant in benign tumours.

**Conclusion:**

The allele with the tandem duplication does not appear to modify breast cancer risk but may act as a low penetrance protective allele for ovarian cancer.

## Background

Fanconi anaemia is a rare autosomal recessive disorder characterised by congenital abnormalities, progressive bone marrow failure, and predisposition to acute myelogenous leukemia and other malignancies. At the cellular level, the disease is characterised by an inability to repair cross-linked DNA [1]. There are multiple genes in which mutations can give rise to Fanconi anaemia.

The *FANCA *gene is defective in more than 65% of Fanconi anaemia cases. *FANCA *and five other Fanconi anaemia genes code for components of a complex that is required for the ubiquitination of FANCD2 in response to DNA damage. Ubiquitinated FANCD2 is targeted to nuclear foci of DNA repair proteins including BRCA1 and RAD51 (reviewed in [2]). The complex also interacts directly with the FANCD1 protein [3]), now known to be the product of the breast and ovarian cancer predisposition gene, *BRCA2 *[4]. As individuals heterozygous for *BRCA2 *mutations have a high lifetime risk of acquiring breast and ovarian cancer, it is likely that alterations in other Fanconi anaemia genes might be associated with an increased risk of breast and ovarian cancer.

In human breast and ovarian cancers, recurrent loss of heterozygosity has been shown to occur on the long arm of chromosome 16 [5,6] and is localised to 16q24.3 [7-9]. This suggests the presence of one or more tumour suppressor genes in this region. However, no recurrent tumour-specific mutations in any of the 16q24.3 candidate tumour suppressor genes assessed for mutations have been reported in breast tumours (e.g. [10-12]).

*FANCA *localises to 16q24.3 [13] and is a plausible tumour suppressor gene candidate because of its role in the repair of DNA damage. Cleton-Jansen et al [14] did not detect any mutations in 19 cases of breast cancer with 16q24.3 loss of heterozygosity and concluded that *FANCA *was not the tumour suppressor gene underlying 16q24.3 loss of heterozygosity. Nevertheless, *FANCA *remains an attractive candidate as either a cancer predisposition gene or a target of genetic or epigenetic inactivation in sporadic tumours.

While screening the *FANCA *promoter region by single strand conformation analysis, we identified a polymorphism in the *FANCA *promoter region. As promoter polymorphisms can alter the transcription or regulation of a gene, we sought to determine whether one of these two alleles might be associated with an altered risk of developing breast or ovarian cancer.

## Methods

### Subjects

All breast cancer cases were systematically ascertained through breast clinics in the Wessex region of southern England as described previously [15,16] These cases were selected on the basis of an age at onset under 40 years, a family history of breast/ovarian cancer (defined as two or more cases of breast/ovarian cancer in a first or second degree female relative) or bilateral breast cancer irrespective of family history or age at onset. Family histories were verified as far as possible from medical records and death certificates. Blood was taken from all recruits who consented to molecular analysis for breast cancer predisposition genes. The age range of the breast cancer participants was 19–76 with a mean age of 40 years

Details of the ovarian tumour cases have been described previously [17,18]. Briefly, cases of ovarian tumours were ascertained from women undergoing primary surgery in hospitals from southern England between 1993–1998. A specialist gynaecological pathologist confirmed the histological diagnosis for each tumour. A total of 390 ovarian tumours were included in the study consisting of 313 malignant (127 serous, 82 endometrioid, 42 mucinous, 13 clear cell, 49 undifferentiated adenocarcinomas), 15 borderline tumours (11 mucinous, 4 serous) and 62 benign tumours (18 fibromas, 26 serous, 18 mucinous). The age range of the ovarian cancer patients was 23–90 with a mean of 62 years.

The controls represent the population from which the cases arose and consisted of 256 Caucasian female volunteers who were either patients attending for non-neoplastic disease conditions or staff at the Princess Anne Hospital, Southampton. The age of the controls ranged from 18 – 84 with a mean age of 39 years. Both control and cancer groups were drawn from the same geographical area. Epidemiological data such as reproductive factors, oral contraceptive use, smoking and obesity were not available for any of the cancer or control groups. We have obtained approval for this research from the appropriate ethics committees and are in compliance with the Helsinki Declaration.

### Molecular genetic analysis

DNA for genotyping was prepared from the peripheral blood samples of patients and controls. The region containing the duplication was amplified using the primers 5'CCAAACGCAAAAACTACCTCACCG3' and 5'CGCTGCCTTCCTATTGGCTGC3'. Fifty ng of DNA was used in a 25 μl reaction using 0.5 U HotStarTaq (Qiagen, Hilden, Germany), 200 nM of each of the primers, 800 μM total nucleotides in the buffer supplied by the manufacturer (Qiagen) and 1.5x Q solution (Qiagen). Cycling conditions were 15 min at 95°C, 11 cycles of 95°C/45 s, 60°-50°C/45 s (decreasing by 1°C per cycle), 72°C/45 s, followed by 34 cycles of 95°C/45 s, 50°C/45 s, 72°C/45 s and finally 10 min at 72°C. The PCR primers amplified a 151 base pair product for allele 1 and a 164 base pair product for allele 2. The products were separated by electrophoresis on 3% agarose gels. PCR products from the three genotypes were sequenced on the ABI Prism 3100 Genetic Analyser (Applied Biosystems, Foster City, CA) using BigDye Terminator v3.1 chemistry (Applied Biosystems).

### Statistical analysis

The Hardy-Weinberg equilibrium was assessed by the standard methods. The data were considered using models assuming dominant inheritance (i.e., women with one or two duplication alleles had the same relative hazard), codominant inheritance (i.e., the relative hazard differed between women with one duplication allele compared with those with two duplication alleles), or recessive inheritance (i.e., only women with two duplication alleles were at increased risk). For all analyses, the control subjects were treated as a single group without stratification. Fisher's Exact test using Instat 3.01 software (Graphpad Software) was used to calculate the significance (*p *value) and odds ratio (OR) with a 95% confidence interval. All statistical calculations were two-sided and *p *values were considered statistically significant when less than 0.05.

## Results

### Identification of the *FANCA *promoter polymorphism

We identified a polymorphism in the *FANCA *promoter region using single strand conformation analysis. Variant bands were purified by PCR amplification from a band stab and sequenced in both directions directly from the PCR product. Comparison of the sequences obtained for each band and the sequence of the *FANCA *promoter (Genbank Accession AC005360) showed that the variation in the patterns was due to the presence of either a single or duplicated 13 base pair sequence.

**Figure 1:**
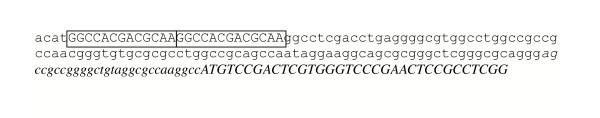
**Sequence of *FANCA *promoter allele in the polymorphic region. **Allele 1 has a single copy of a 13 base pair sequence. This has been defined as GGCCACGACGCAA in accordance with current nomenclature for mutations [21] although it could theoretically be GCCACGACGCAAG or CCACGACGCAAGG. Allele 2 has two tandemly arranged copies of this sequence. The 13 base pair sequence is located at -98 to -110 bases upstream of the beginning of transcription as defined by the NCBI reference sequence NM_000135.1. Exon 1 is in bold and the coding sequence in capitals.

The 13 base pair sequence is located at -98 to -110 bases upstream of the beginning of transcription as defined by the NCBI reference sequence for *FANCA *(NM 000135.1 23-Dec 2003). The translation start site is 32 base pairs further downstream (Figure 1). Allele 1 has a single copy of the sequence GGCCACGACGCAA. Allele 2 has two tandemly arranged copies of this sequence. Interestingly, the sequence immediately downstream of the 13 base pair sequence, GGCCtCGACctgA shows considerable homology to the 13 base pair sequence (divergent nucleotides in lower case).

### Case control study of the *FANCA *promoter polymorphism

**Figure 2:**
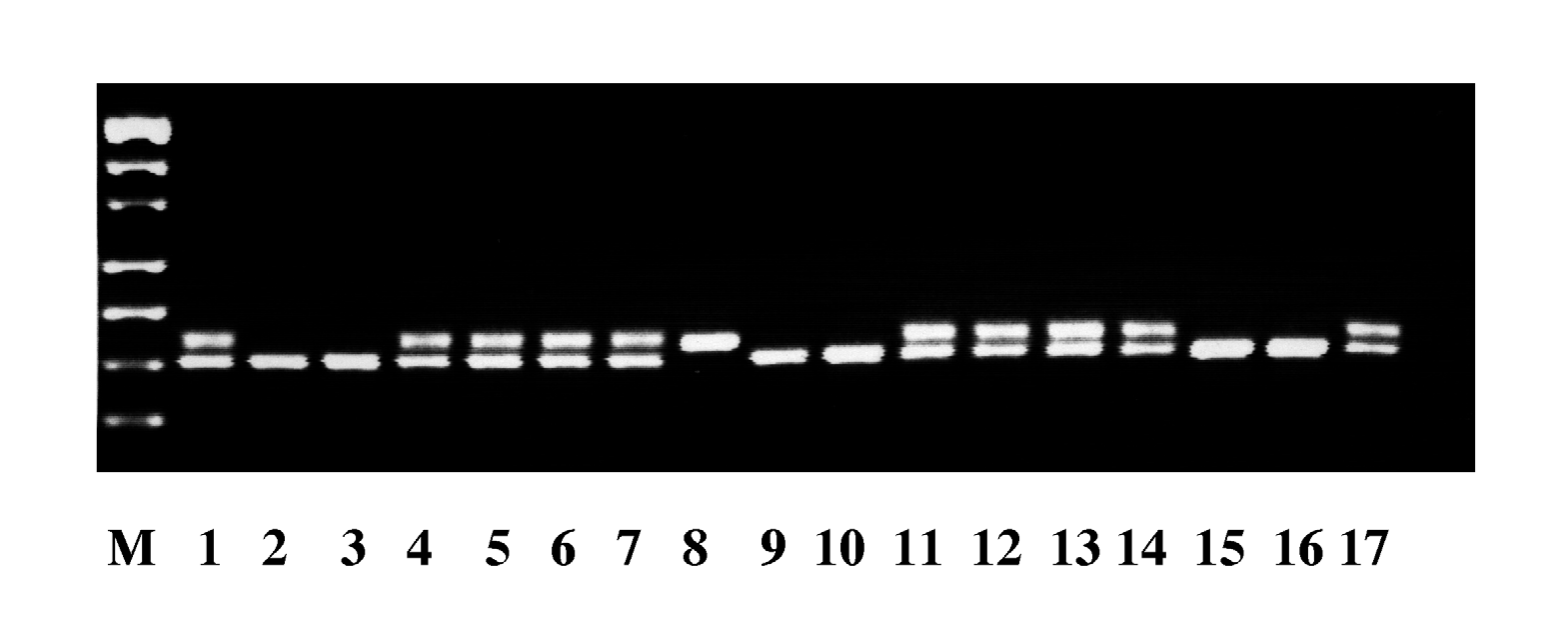
**Genotyping the FANCA promoter polymorphism by PCR. **Allele 1 amplifies as a band of 151 base pair, allele 2 as a band of 164 base pair. The 3 genotypes are readily distinguishable on a 3 % agarose gel run at 100 V for 1 hour. Homozygous samples for both allele 1 and allele 2 are shown as are heterozygous samples which have both bands.

We designed a PCR assay in which both alleles are amplified simultaneously using a set of primers flanking the polymorphism (Figure 2). The frequency of the polymorphism was determined in breast cancer patients, ovarian cancer patients and controls to assess if the presence of either allele was associated with a predisposition to breast or ovarian cancer.

Table 1 shows the distribution of genotypes in the breast and ovarian cancer patients and the controls. The distribution of the genotypes within each of the groups did not deviate significantly from those expected under Hardy-Weinberg equilibrium.

The frequency of the duplication allele (allele 2) in the breast cancer patients was not significantly different from the controls (0.32 versus 0.34, p = 0.53). The distribution of genotypes in the breast cancer cases was similar to the controls. The genotype distribution was similar when stratifying the breast cancers according to the selection criteria although the frequency of the 12 and 22 genotypes showed a non-significant elevation among the family history group.

The frequency of the duplication allele in the ovarian cancer patients was 0.29 which was significantly different from the controls (p = 0.048). In addition, the ovarian cancer patients had a nominally significant decrease in the number of 12 and 22 genotype carriers compared to the controls (p = 0.05) suggesting that the duplication may be protective for the disease with an odds ratio of 0.72 (95% CI, 0.53–0.99). All subgroups of the ovarian cancer patients showed a reduced frequency of 12 and 22 genotypes, with the exception of patients with borderline tumours and clear cell carcinomas. However, the number of cases in these latter categories was very small. Interestingly, the 62 patients with benign tumours showed a highly significant decrease in the 12 and 22 genotype frequency (p = .007) with an odds ratio of 0.46 (95% CI 0.26–0.81).

## Discussion

The identification of *BRCA2 *as a member (*FANCD1*) of the Fanconi anaemia group of genes has raised the possibility that variation in other Fanconi anaemia genes may predispose to breast or ovarian cancer. The novel 13 base pair duplication allele identified in this study is of interest as even single base changes in promoter sequences can alter regulation of gene expression and contribute to tumorigenesis, particularly if this affects a transcription factor binding site (e.g. [19,20]). As both alleles of the *FANCA *polymorphism are common, it is unlikely that either allele would represent a high penetrance predisposition allele. We therefore undertook a case control study to determine whether either allele might be responsible for a more modest increase in the rate of breast and or ovarian cancer.

Genotyping of the promoter polymorphism in high risk breast cancer patients showing features of genetic predisposition revealed no significant difference in the allele or genotype distribution compared to the normal controls. The study had 80% power to detect an odds ratio of ≥ 1.6 for carriers heterozygous for the duplication and an odds ratio ≥ 1.9 for carriers homozygous for the duplication. As we could not adjust for known breast cancer risk factors, we cannot exclude the possibility that confounding factors may have led to a type II error. However, confounding due to differences in ethnicity is unlikely as both the cases and controls were residents of the Southampton area, which has a predominantly Anglo-Saxon population. Nevertheless, studies of breast cancer cases selected based on other clinical characteristics, such as postmenopausal onset, may be warranted.

Among the ovarian tumour cases, there was a significant decrease in the frequency of the combined 12 and 22 genotypes, suggesting that allele 2 may protect against ovarian cancer. The trend was particularly evident among the benign tumours (p = .0007). However, it is possible that this association represents a type I error due to confounding factors such as ethnicity and the influence of known risk factors such as oral contraceptive use. Consequently, it will be important to replicate our findings in larger population-based case-control studies.

## Conclusion

We have identified a novel promoter polymorphism in *FANCA*. It is unknown what biological effect the duplication might have but it may alter the basal rate of transcription or the regulation of transcription. As *FANCA *is likely to function as a tumour suppressor, it is plausible that allelic variants with altered activity may modify cancer risk. This pilot study has provided some evidence of an association with ovarian cancer. Further studies to verify this association are warranted as well as studies involving predisposition to other cancers.

## Competing interests

The author(s) declare that they have no competing interests.

## Authors' contributions

ET performed the genotyping of the breast and ovarian cancer patients and controls. RD identified the polymorphism and did the initial breast cancer patient genotyping. ET and RD contributed equally to this work. SS was involved in planning through the course of the project and helped prepare the manuscript. DE collected the patients used in this study. IC provided the DNA samples, participated in shaping the study and analysed the results. AD conceived the study, supervised the research and prepared and revised the manuscript.

## Pre-publication history

The pre-publication history for this paper can be accessed here:



## Figures and Tables

**Table 1:** The distribution of FANCA promoter polymorphism genotypes in breast and ovarian cancer cases and controls

**Study Group**	**No.**	**11**	**12**	**22**	**12 + 22**		
		
		**n (%)**	**n (%)**	**n (%)**	**n (%)**	**p value^a^**	**OR^b ^(95% CI)**
**Control**	**256**	**112 (43.8)**	**115 (44.9)**	**29 (11.3)**	**144 (56.2)**		
**All Breast Cancer**	**352**	**164 (46.6)**	**150 (42.6)**	**38 (10.8)**	**188 (53.4)**	**0.51**	**0.89 (0.64–1.23)**
Diagnosis ≤ 40 years	203	104 (51.2)	79 (38.9)	20 (9.9)	99 (48.8)	0.13	0.74 (0.51–1.07
Family History	105	39 (37.1)	53 (50.5)	13 (12.4)	66 (62.9)	0.29	1.32 (0.83–2.10)
Bilateral Disease	44	21 (47.7)	18 (40.9)	5 (11.4)	23 (52.3)	0.63	0.85 (0.45–1.62)

**All Ovarian Tumours**	**390**	**202 (51.8)**	**153 (39.2)**	**35 (9.0)**	**188 (48.2)**	**0.05**	**0.72 (0.53–0.99)**
All Malignant	313	157 (50.2)	126 (40.2)	30 (9.6)	156 (49.8)	0.13	0.77 (0.55–1.08)
Serous	127	62 (48.8)	52 (40.9)	13 (10.2)	65 (51.1)	0.38	0.81 (0.53–1.25)
Endometrioid	82	44 (53.6)	29 (35.4)	9 (11.0)	38 (46.4)	0.13	0.67 (0.41–1.11)
Mucinous	42	23 (54.8)	16 (38.1)	3 (7.1)	19 (45.2)	0.24	0.64 (0.33–1.24)
Clear Cell	13	4 (30.8)	8 (61.5)	1 (7.7)	9 (69.2)	0.40	1.75 (0.52–5.83)
Adenocarcinoma	49	24 (49.0)	21 (42.8)	4 (8.2)	25 (51.0)	0.53	0.81 (0.44–1.49)
Borderline	15	6 (40.0)	8 (53.3)	1 (6.7)	9 (60.0)	1.00	1.16 (0.40–3.37)
Benign	62	39 (62.9)	19 (30.6)	4 (6.4)	23 (37.0)	0.007	0.46 (0.26–0.81)

Allele 1 corresponds to the single copy allele, allele 2 corresponds to the duplication allele.

^a^Fisher's exact test (two-sided) for the combined 12/22 genotype frequency using the 11 homozygotes as reference.

^b^The odds ratio (OR) and 95% confidence intervals (CI) are shown in parentheses.
